# The seasonal movement of sediment-associated marine-derived nutrients in a morphologically diverse riverbed: the influence of salmon in an Interior British Columbia river

**DOI:** 10.1007/s11368-023-03563-2

**Published:** 2023-06-21

**Authors:** Kristy A. Rasmus, Ellen L. Petticrew, John Rex

**Affiliations:** 1 Ministry of Environment and Climate Change Strategy, 3726 Alfred Street, Smithers, BC V0J 2N0 Canada; 2 Department of Geography, Earth and Environmental Sciences, University of Northern British Columbia, 3333 University Way, Prince George, BC V2N 4Z9 Canada; 3 Ministry of Forests Omineca Region, Research and Forest Health, 5th Flr. 499 George St., Prince George, BC V2L 1R5 Canada

**Keywords:** Sediment, Salmon, Marine-derived nutrients, MixSIAR, Flocculation

## Abstract

**Purpose:**

This study (1) investigated the extent to which flocculation and the hydrological and morphological attributes of an interior salmon-bearing river regulate the seasonal storage of marine-derived nutrients (MDN) and (2) compared the contribution of MDN to the fine bed sediment relative to other nutrient sources to the river.

**Methods:**

Previous research has determined that the co-existence of re-suspended fine sediment, generated by salmon redd construction, with salmonid excretion and decay products in the water column creates ideal conditions for the flocculation of these inorganic and organic particles. Stored and suspended fine bed sediment was sampled from seven sites with varying morphologies and bed substrate down the length of a large spawning river in the interior of British Columbia over a 12-month period. MDN contributions to the sediment was tracked using aggregated versus dispersed particle size, carbon-to-nitrogen ratios, stable carbon and nitrogen isotopes, and MixSIAR modeling.

**Results and discussion:**

(1) There was a significant longitudinal spatial distinction of nutrient retention between sites upstream and downstream of a large seasonally inundated floodplain; (2) the MDN isotopic signal in the surficial stored bed sediment in this sample year was short term; and (3) upstream spawner numbers, substrate size, stream morphology, and discharge were relevant to both the magnitude and retention time of sediment-associated MDN.

**Conclusion:**

A cumulative magnification of MDN was correlated with the distance from the headwaters and the number of upstream spawners. The relationship between MDN retention in interior rivers, and possible multi-year accumulation, was influenced by variability in channel morphology, substrate size, and the presence of an inundated floodplain halfway down the river.

## Introduction

Sediment in aquatic ecosystems is most often discussed in terms of the negative impacts of excessive loading from terrestrial sources, such as hydro-electric dam construction, urban and road development, forest wildfire or harvest, or mass wasting events (Waters 1995; McAdams et al. 2005; Eaton et al. 2010; Owens 2020). Fine sediments (< 63 μm diameter) introduced into a stream channel in excess of background level can clog interstitial spaces in gravel beds, thus reducing the flow of oxygen to benthic biota (Wood and Armitage 1997; Wharton et al. 2017) and fish eggs (Malcolm et al. 2004; Jensen et al. 2009). Although excess fine sediment can be a pollutant, in a natural system under natural levels, fine sediment acts as a vector to move organic matter, or nutrients, down the stream gradient. Most modeling of sediment delivery within a river assumes individual particles, but many move as aggregate particles or flocculants (a.k.a. flocs). The development of these composite particles involves the aggregation or flocculation of fine sediment and organic matter. A wide range of freshwater hydrological, chemical, and biological conditions favor the formation of flocs (Droppo et al. 1997). Natural aquatic flocs have both a biotic (microorganisms, detritus, cellular material) and abiotic (clays and silts) component and are abundant in both fresh and saltwater environments. Bacteria found on the surface of organic matter have extracellular polymeric substances that act as a “glue” between the organic and inorganic components. Therefore, the source of the sediments and organic matter, along with the associated bacteria regulate the extent of flocculation (Droppo et al. 2015). This variation in organic matter quality thereby influences the resulting floc size, shape, and settling behavior (Petticrew and Arocena 2003), which act to determine the fate of these flocs and the effect of their associated nutrients in natural systems.

Interior streams in British Columbia (B.C.) receive most of their sediment and nutrient inputs as pulses of upstream terrestrial riparian vegetation, bank and riparian erosion, and the re-mobilization of in-stream subsidies (Wipfli et al. 2007). In this way, the nutrient dynamics of an interior stream are largely controlled by the hydrological processes interacting with channel riparian structure and morphologies that regulate the balance between delivery, mobilization, and storage (Droppo 2001; Owens et al. 2005). Spawning Pacific salmon, however, are also capable of re-mobilizing substantial quantities of fine bed sediment while cleaning the gravels to prepare their egg nests known as redds (Hassan et al. 2008; Moore and Schindler 2008). The difference though between salmon and hydrological flushing events is that first, spawning typically occurs during periods of low flows and disturbance is not evenly distributed throughout a stream, and second, spawning salmon also contribute a large pulse of dissolved and particulate marine-derived nutrients (MDN) both from live salmon excretions and decaying salmon tissue which extends past the time when active redd building is occurring (Gende et al. 2002). Marine-derived refers to the fact that approximately 95% of salmon body mass is accumulated in the marine environment (Naiman et al. 2002). Therefore, Pacific salmon returning from the ocean to spawn, die, and decay in their natal interior and coastal freshwater streams transfer external nutrients, and thereby create ideal conditions for flocculation between fine sediment and organic matter.

Previous MDN research has focused primarily on trends in the contribution of soluble nitrogen and phosphorus (Mitchell and Lamberti 2005; Cak et al. 2008; Collins et al. 2011). The processes regulating particulate-associated MDN may act to retain (by settling) and retard (through gravel bed storage, biological uptake, and eventual breakdown) the downstream movement of MDN. Controlled flume experiments identified that fine sediment, such as that re-suspended by spawning salmon, adhered with organic matter from decaying carcasses to form flocs within 20 m of nutrient and sediment inputs (Petticrew et al. 2011). In a laboratory setting, Arkinstall (2005) found that decaying salmon matter produced larger and faster settling flocs than breakdown products from algal matter. Once on the streambed, MDN can then be entrained in biofilm and/or transported into the substrate interstitial zones (Johnston et al. 2004; Tiegs et al. 2008). Substrate biofilm (a.k.a. periphyton) includes the attached algal community and the associated microbes, bound organic matter, and trapped inorganic fine sediment. The synchronous supply of floc-forming components of inorganic fine sediment and salmon decay products suspended in the river column was called the salmon disturbance regime by Albers and Petticrew (2012). They hypothesized that it was a mechanism for in-stream retention of MDN as documented by Rex and Petticrew (2008).

Most of the previous research on the release of MDN and salmon disturbance effects has focused on coastal rivers, often restricted to localized spawning reaches. The timing and duration of salmon spawning, and the concomitant nutrient dynamics, along with flow regimes and therefore fine sediment delivery, differ between interior and coastal streams. The spawn event in interior streams typically occurs later in the summer than coastal streams, and therefore closely precedes the natural decrease in primary productivity due to reduced daylight and colder air and water temperatures. Interior streams have snowmelt driven hydrological regimes and discharge through the winter months which is typically low and steady, whereas coastal streams are rain dominated and experience higher and more variable post-spawn discharge. The potential for MDN to significantly contribute to primary productivity in interior streams, both within the stream itself as well as in the downstream nursery lakes, may therefore depend more on the location and availability of MDN during the following spring and summer months, rather than solely during the spawn event (Fellman et al. 2008). The aim of this project was to investigate the role of surficial fine bed sediment in the seasonal movement of MDN over the length of a large interior river, including spawning and non-spawning reaches. Thus, downstream changes in habitat complexity and the dynamics of longitudinal spiraling processes are important considerations, especially when studying large structurally variable interior watersheds, and cannot be addressed by investigating singular spawning reaches. The length of time sediment-associated MDN is retained on the streambed surface or within the gravel bed, and the combination of physical stream characteristics that facilitate the retention is poorly understood, but both depend in the broadest sense, on the same riverine conditions (for example, discharge, local shear, bed structure, and water temperatures) that control the uptake and release of nutrients and organic matter by aquatic organisms and hydrological scouring of the benthic environment (Tiegs et al. 2008; Albers and Petticrew 2013; Joy et al. 2020). In the face of a changing climate that is altering watershed hydrology and impacting salmon populations, being able to better predict how MDN availability, regulated by the relationship between flow and channel complexity, is imperative for understanding the potential impact on future salmon stocks and informing adaptive stream management.

The objectives of this study were, therefore, to investigate: (1) the extent to which flocculation was associated with the storage of MDN; (2) the relationship between seasonal movement of MDN and the hydrological and morphological attributes of the river; and (3) the contribution of MDN to the surficial fine bed sediment relative to other riverine nutrient sources.

## Methods

### Study area

**Fig. 1:**
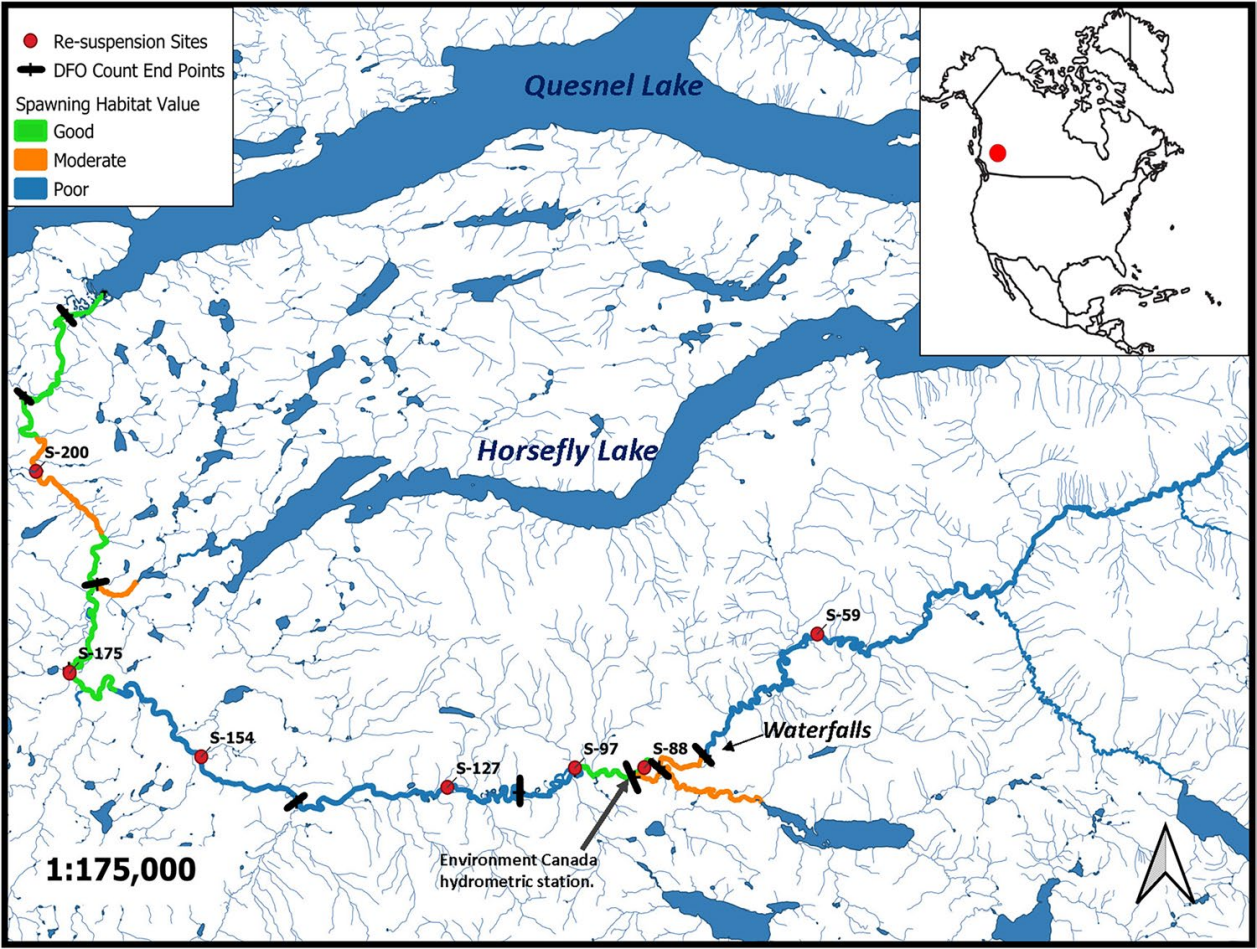
Map of the location of the Horsefly River including study sites, river-gauging positions (BC Ministry of Environment and University of Northern British Columbia installed), and Canadian Federal Government, Department of Fisheries and Oceans (DFO) characterization of spawning habitat quality and salmon count river reach end points

The Horsefly River is part of the Fraser River basin and is located in the Cariboo region of Central Interior British Columbia. The river runs from the headwaters in the Cariboo Mountains through the town of Horsefly and drains into Horsefly Bay on Quesnel Lake (Fig. 1). The Horsefly River is 215 km long, has a Strahler stream order of 6, and is fed by a 2765 km^2^ watershed (British Columbia Watershed Atlas 2011). The flow regime is snowmelt dominated and therefore exhibits high seasonal variability, with historical mean peak discharges of approximately 80 m^3^ s^−1^ and historical mean base flows of approximately 10 m^3^ s^−1^.

### Sampling design and site characteristics

From August 11, 2014 to August 20, 2015, 279 fine sediment samples (< 63 µm) stored in (top 10 cm) and on the surface of the channel bed (hereafter called surface sediments), and 13 suspended sediment samples were collected from seven sampling sites during 25 sampling events. The seven sites were spread relatively evenly over approximately 140 km of the Horsefly River (Fig. 1) and represented a range of channel conditions such as sediment depositional and active flow morphologies, and bed compositions. An extensive floodplain exists between approximately 110 km and 135 km, and although it is referred to in this paper as a floodplain, it is inundated for about a month each spring and therefore functions as a shallow lake during and following the snowmelt.

The most upstream site, S-059 (59 km downstream of headwaters), was located approximately 20 km above a set of falls that are a natural salmon barrier. The furthest downstream site, S-200, was 200 km downstream of the headwaters and approximately 15 km upstream of Horsefly Bay on Quesnel Lake, the salmon nursery grounds. Sampling sites varied by flow morphology, substrate size, and quality of spawning habitat. Substrate at each site was classified using Wentworth substrate classification groups and the *D*_50_ as determined from Wolman pebble counts (Wolman 1954). Table 1 lists the physical attributes of the individual sample sites. River stage and discharge were recorded at an Environment and Climate Change Canada (ECCC) hydrometric gauging station located just downstream of S-088 (Fig. 1). River stage and water temperature were also recorded using HOBO Onset pressure transducers near S-127 and between S-200 and the mouth of the river.

**Table 1:** Description of physical attributes for sampling sites along the Horsefly River. D_50_ is the median substrate size in millimeters based on a 100-pebble count. Wentworth class is the classification of substrate size based on the pebble count

**Site**	**Spawning habitat**	***D***_**50**_ (mm)	**Wentworth class**	**Morphology**
**S-059**	Above salmon barrier	NA	Coarse gravel	Pool + eddy-run (depositional)
**S-088**	Good spawning habitat at site, and ~7 km of moderate spawning habitat immediately upstream	29	Coarse gravel	Riffle-run
**S-097**	Moderate spawning habitat at site, ~18 km of good spawning habitat upstream between 088 and 097	22	Medium gravel	Pool + eddy-run (~10 km upstream of freshet-inundated floodplain; depositional
**S-127**	No spawning habitat at site, poor spawning habitat upstream between 097 and 127	< 2	Sand	Meander (within the freshet-inundated floodplain; depositional)
**S-154**	No spawning habitat at site, poor spawning habitat upstream between 127 and 154	110	Medium cobble	Eddy-run (~19 km downstream of freshet-inundated floodplain)
**S-175**	Good spawning habitat at site, and ~7 km of good spawning habitat immediately upstream of site	46	Coarse gravel	Eddy-run
**S-200**	~30 km of good to moderate spawning habitat between 175 and 200	26	Coarse gravel	Eddy-run

### Bed sediment collection and analysis

The collection of fine-grained, channel-stored bed sediment involved re-suspending the top 10 cm of bed substrate within an enclosure using a large open-bottom container of known volume (truncated cone). This method is similar to that first described by Lambert and Walling (1988), and more recently reviewed by Duerdoth et al. (2015). Ten to 15 L of water, depending on turbidity (i.e., visual estimate of suspended sediment concentration), was removed from the measured volume of water in the enclosure into a 20 L (5-gallon) bucket for further laboratory separation. While this is termed surficial sediment, in this paper it represents the material settled onto the gravel bed as well as that stored in the matrices of the top 10 cm of gravels. This sample included fine benthic sediment and dislodged large but light particles of biofilm as the bounded water column was allowed to settle for 10 s (sand settles at  ~1 cm per second) to ensure heavier/fast settling particles, including pebbles and sand, were not collected. Re-suspension of the channel bottom was repeated at two positions within 5 m from one another at each sample site. Due to the width/depth of the Horsefly River, all samples except for one at S-059 and one at S-097, which had accessible mid-stream bars, were taken within 2–3 m of the wetted edge. From May 14 to June 18 of 2015, channel bed re-suspensions were not possible due to the elevated water levels, and therefore bulk suspended sediment was collected and transported back to the lab in and settled in four to eight 20 L buckets. Only three to four of the sites were sampled during each event due to transport capacity and access during the flood. These sites included, S-059 each time, S-088 or S-097, and S-154, S-175 or S-200. Processing the channel-stored sediment involved taking two 50 mL sub-samples following mixing of the bucket sample in the laboratory, then leaving the remainder of the sediment to settle overnight before the excess water was siphoned off. The bulk sediment collected after siphoning was stored at −20 °C until further preparation for nutrient and stable isotope analysis. One of the 50 mL sub-samples was filtered through a pre-ashed Whatman^®^ glass-fiber filter. The filter with sample was then dried for 24 h at 60 °C and ashed for 1 h at 550 °C. The dry and ashed weights were used to calculate the total grams of sediment per m^2^ of riverbed and the ratio of organic to inorganic matter (OMR) (Hauer and Lamberti 2011). The second 50 mL sub-sample was analyzed for particle size characteristics including aggregated and dispersed particle size distributions and summary parameters of *D*_10_, *D*_50_, *D*_90_, and specific surface area (SSA = m^2^ kg^−1^). Particle size analysis (PSA) was undertaken on a Malvern Mastersizer 3000^®^. PSA samples were stored in the dark at 4 °C to reduce biological degradation and aggregation alterations. Samples were considered dispersed after being sonicated in the Malvern for 2 min at 90% power (45W) (Koiter et al. 2013). The “floc factor” was calculated by dividing the aggregated *D*_50_ by the dispersed *D*_50_ (McConnachie and Petticrew 2006).

### Source material collection and stable isotope analysis

Organic source material that was determined to contribute to the stream loadings were collected from both the channel and riparian areas. Samples of salmon tissue, muscle, and skin were collected throughout the entire study area from dead fish. One whole salmon carcass was included to collect organ and egg samples. Fifteen non-salmon nutrient sources were also sampled. Non-salmon source material included live (summer) and dead (fall) riparian vegetation and aquatic vegetation (vascular macrophytes) (Table 2).

**Table 2:** A list of source material included in each source group used in statistical mixing models, along with the number of samples collected for each source

**Source group**	**Source Material**	**# of samples**
**Sockeye salmon**	Muscle and skin tissue	8
	Organ tissue	1
	Eggs	1
**Aquatic macrophytes**	Aquatic buttercup (*Ranunculus aquatilis*)	2
	Tapegrass (*Vallisneria americana*)	2
	Pondweed (*Potamogeton* spp.)	5
	Waterweed (*Elodea* spp.)	5
**Riparian vegetation**	Horsetail (*Equisetum arvense)*	6
	Moss (*Bryophyte spp.)*	4
	Grass (*Carex* spp.)	5
	Willow (*Salix* spp.)	4
	Alder (*Alnus sitka)*	4
	Red-osier dogwood (*Cornus stolonifera)*	4
	Spirea (*Spirea* spp.)	1
	Black cottonwood (*Populus balsamifera)*	3

Sediment samples (surface channel bed and suspended) and source material were analyzed for C and N isotopes and percent of C and N with an elemental analyzer interfaced to a continuous flow isotope ratio mass spectrometer (IRMS) at the University of California Davis Isotope Lab. Isotopic enrichment was expressed as δ^13^C or δ^15^N, which refers to the deviation values from a standard in parts per thousand (Hauer and Lamberti 2011). Delta values are determined as follows:

1 $$\updelta \mathrm{X}\left({\updelta }^{{13}}\;\mathrm{C\;or}\;\updelta ^{\mathrm{15}}\mathrm{N}\right)=[({\mathrm{R}}_{\mathrm{sample}}-{\mathrm{R}}_{\mathrm{standard}}){/{\mathrm{R}}_{\mathrm{standard}}}]\times \mathrm{1000}$$

where R is the ratio of the heavy isotope to the light isotope (^15^N/^14^N or ^13^C/^12^C). The standard for C is Peedee Belemnite and air for N (Bilby et al. 1996). The %N and %C values were used to determine the molar ratios of C to N (C:N).

The δ^15^N determined from the above calculation was used to back-calculate the percentage of N in the sample that was ^15^N. This value was used to calculate MDN load as grams of ^15^N per m^2^ of riverbed using the following equations:

2 $${}^{15}\mathrm{N\%\;of\;total\;N} =100-100/(0.003676 (1+{\delta }^{15}\mathrm{N}/1000))+1$$

$$\begin{aligned}\mathrm{Mass}\;&\mathrm{of}\;^{15}\text{N}(g)/g{\;\mathrm{of}\;\mathrm{sediment}}\\&=(({}^{15}\mathrm{N}{\% }{\;\times\;\mathrm{Total}}\;g{\;\mathrm{N} \;\mathrm{in}\;\mathrm{sample}}\;/ {\;\mathrm{Total}}\;g{\;\mathrm{of}\;\mathrm{Sample}})\end{aligned}$$

$$\begin{aligned}{\mathrm{Mass}\;\mathrm{of}\;{}^{15}\mathrm{N}}&(g){/{\mathrm{m}}^{2}\;\mathrm{of}\;\mathrm{the}\;\mathrm{bed}\;\mathrm{substrate}}\\&=({g}^{15}\mathrm{N}/g{\;\mathrm{of}\;\mathrm{sediment}\;\mathrm{collected}})/{}^{*}0.0871 {{\mathrm{m}}^{2}}\end{aligned}$$

$${}^{*}0.0871 {m}^{2}\;is\;area\;of\;substrate\;sampled\;under\;sampling\;bucket$$

### Statistical analysis

Statistical analysis was used to interpret spatial and temporal patterns and proportional contributions of MDN in the bed sediment. Spatial analysis looked at the relationships between sampling sites and temporal analysis looked at the relationships between sediment collected during different seasons. Five seasonal groupings were made based on natural seasonal changes in the interior of B.C., as well as the presence and absence of spawning salmon in the river. The five seasons and the corresponding months sampling dates are: pre-spawn (August 2014); spawn (September to mid-October 2014); post-spawn (late October to December 2014 + late March to mid-April 2015); freshet (late April to mid-June 2015); and post-freshet (July to September 2015).

#### Spatial and temporal analysis of sediment MDN

Linear mixed-effects models were used to compare the effect that season, sampling site, channel morphology, and substrate size had on the response of sediment δ^15^N and MDN load. For these models, season included samples grouped into pre-spawn, spawn, post-spawn, and post-freshet. Suspended sediment collected during the freshet was not included in this analysis because only a subset of sites was sampled, and due to the mixed nature of the suspended material its source location substrate or morphology class was unknown. Sediment collected at the site located above the fish-barrier waterfalls (S-059) was only included in models when sample site was an explanatory variable. Models with sites grouped into channel morphology (eddy, riffle/run, and pool) or substrate size (coarse gravel, medium gravel, cobble, and sand) did not include S-059. Sample site and sample date were included in each model as random effects to account for the repeated measures sampling design. Fixed effects were evaluated for normality, homogeneity, and spatial independence. Non-normal data were log (base 10) transformed. AIC values were used to compare models, and *R*^2^ values for the fixed and random effects was used as a guideline for how well each model estimated the amount of variation the explanatory variables predicted (Nakagawa and Schlielzeth 2013). A modified pairwise Tukey test was used as a guide to further analyze model results. Statistical analysis was done using R statistical software (Bates et al. 2015; Kuznetsova et al. 2017; Barton 2020; v4.0.3; R Core Team 2020; Lüdecke 2021;v4.0.3; Russel 2022).

The relationship between sediment MDN and upstream salmon spawner density plus distance from river headwaters were analyzed using simple linear regressions. Data for spawner density was estimated from spawning numbers provided by the Department of Fisheries and Oceans (DFO). Over the course of the 2014 spawn DFO technicians counted a subset of sockeye salmon in the river at nine sampling sections (pers. comm. with Brian Leaf, DFO). The end points for each of the DFO count sections are shown in Fig. 1. The final count reported by DFO was 457,553 spawners in the entire Horsefly River (NuSEDS DFO site). The number of salmon counted within each section was used to calculate the percentage of salmon upstream of each of the re-suspension sampling sites (Table 3).

**Table 3:** Sockeye salmon counts provided by the Canadian Federal Department of Fisheries and Oceans (DFO). Distance between sites was determined using from spatial data obtained from the BC Watershed Atlas using ESRI ArcGIS. Horsefly Bay (HFB) is the mouth of the river on Quesnel Lake. All values are cumulative to include complete upstream quantities

**Sample site**	**Percent of total sockeye upstream of site (%)**	**Total cumulative # of upstream sockeye**	**Distance to closest upstream site (km)**
**S-059**	0.00%	0	
**S-088**	1.63%	7458	29
**S-097**	10.14%	46394	9
**S-127**	30.01%	137306	30
**S-154**	31.47%	143986	27
**S-175**	48.86%	223551	21
**S-200**	63.61%	291037	25
**HFB**	100.00%	457533	20

#### Seasonal contribution of salmon to the bed sediment

The seasonal contribution of spawning sockeye salmon to the bed sediment was estimated with MixSIAR, a Bayesian mixing model created for use in the statistical software R (Ward et al. 2010; Stock and Semmens 2013). The Bayesian framework of MixSIAR fits models hierarchically using a Markov Chain Monte Carlo (MCMC) method (Plummer 2021), and can include uncertainty in the sources used, such as differing elemental concentrations and trophic enrichment as well as incorporating fixed and random effects (Semmens et al. 2009; Parnell et al. 2013). The “mix” in the model was the sediment sampled at each site below the falls where salmon were present. Bed sediment sampled from above the falls, at S-059, and suspended sediment sampled during the freshet were not included in the mixing model.

The 15 source materials were grouped into three categories, riparian vegetation (shrub and deciduous tree leaf litter plus non-woody vascular plants), aquatic vascular macrophytes, and sockeye salmon tissue (Table 2). These groupings were initially determined by assessing vegetation classifications and method or timing of input to the river and later confirmed by comparing the isotopic biplots of ^15^N and ^13^C distributions. Reducing the number of sources strengthens the mixing model by limiting the number of overlapping source distributions (Phillips and Gregg 2003; Parnell et al. 2010).

No corrections for trophic enrichment were used in the models because the level of enrichment or discrimination occurring in the biofilm is random. Stock and Semmens (2016) suggested that sediment fingerprinting mixing models would likely follow the model assumption that sediment as a consumer is a “perfect integrator, but with residual error.” MixSIAR differs from earlier mixing models by including an option of correcting for concentration dependence biases. Concentration dependence is a concern if the elemental concentrations of the source materials differ significantly and/or if the consumer metabolizes C and/or N differently (Phillips and Koch 2002). Using Bayesian mixing models for sediment fingerprinting is a relatively new practice. This study combines elements from both sediment fingerprinting and food web models. The end point or “consumer” in this case is sediment, but the source material is organic matter similarly used in food web models. Correcting for concentration dependence is an accepted practice in food web models and is also becoming more common for sediment fingerprinting models (Upadhayay et al. 2017; Reiffarth et al. 2019). We chose to correct for concentration dependence in this model due to the differences in elemental concentrations between the N-rich salmon tissue and the relatively N-poor plant matter. MixSIAR incorporates concentration dependence in the model by adding an argument (i.e., a table) with values for %C and %N for each source material. The models were run using the very long chain length (i.e., 1,000,000 iterations).

## Results

### Trends in discharge and sediment characteristics

**Fig. 2:**
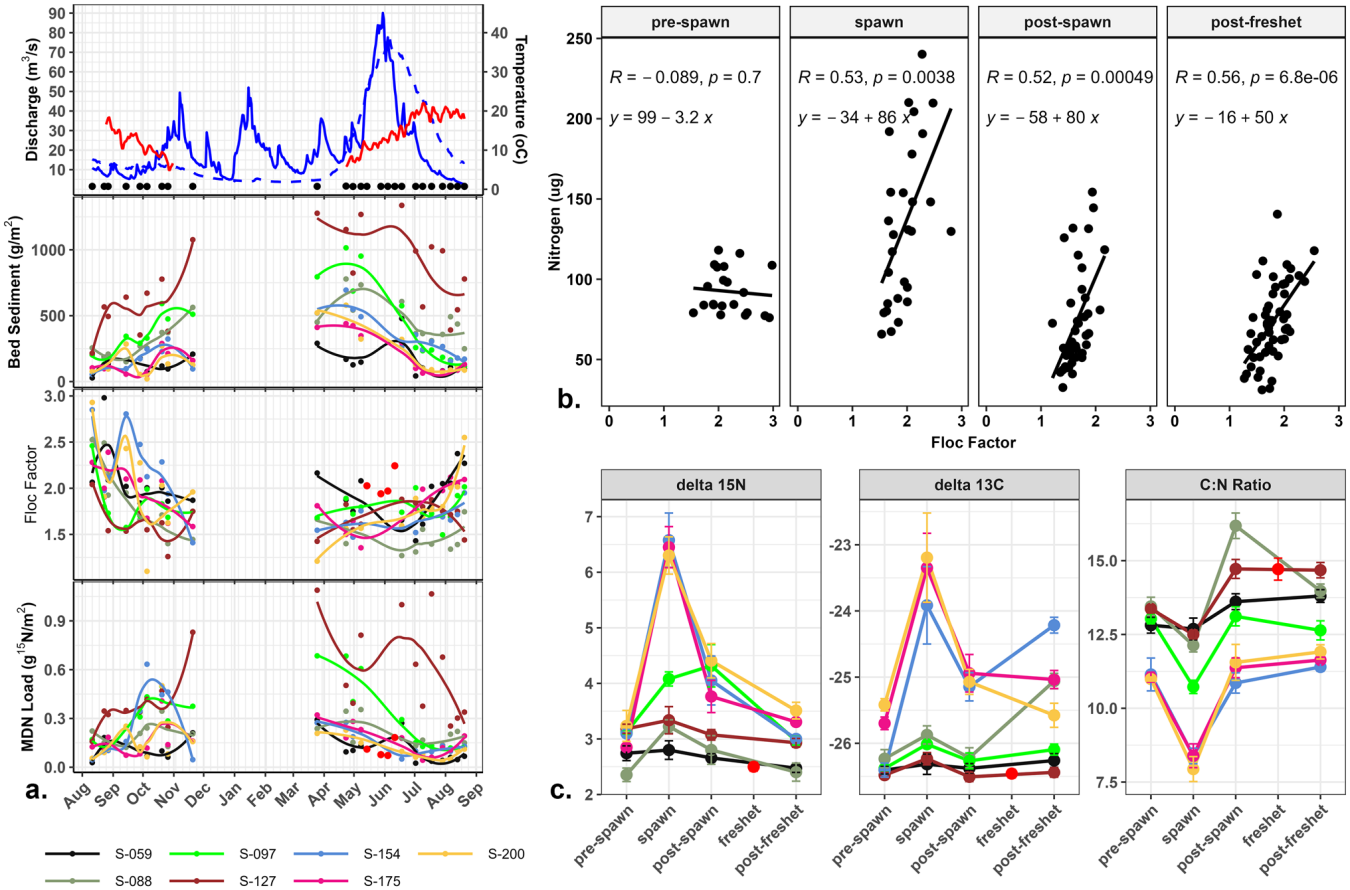
**a** Discharge data (blue) from the Environment and Climate Change Canada hydrometric station at McKinley Creek (historical average discharge in hatched blue) and river temperature (red) recorded from an Onset Tidbit installed ~5 km upstream of Horsefly Bay. The black dots on the hydrograph represent each sampling event. The seasons labeled on the hydrograph relate to groupings for the linear mixed-effects models. The light-gray area within the spawn represents peak-spawn. For easier visualization of trends at all sites, the values for g m^−2^ of fine bed sediment (on and up to 10 cm depth) and MDN load at only S-127 were halved. **b** Scatter plots showing the linear correlation between the floc factor (ratio of aggregated sediment to disaggregated sediment) and the amount of sediment-associated nitrogen (µg). **c** Plots of mean values with one standard error bar for sediment nutrient characteristics during each season

Sediment sampling began mid-August of 2014, and the first sockeye salmon were observed moving up the Horsefly River in late August. The Department of Fisheries and Oceans (DFO) reported that peak-spawn (when > 50% of salmon in river are spawning) occurred from September 6 to 20 (pers. comm. Tracey Cone, DFO). By October 20, no live salmon remained in the river and by October 26 only the remnants of a few carcasses were observed. River discharge was obtained from the ECCC hydrometric station located just below site S-088, approximately 130 km upstream of the river mouth (Fig. 1). Discharge and river temperature are plotted along with the quantity of surface bed sediment (g m^−2^), the floc factor, and MDN load (g ^15^N m^−2^) in Fig. 2a. River temperature steadily declined from approximately 15 °C at the beginning of the salmon run to 6–9 °C by the end of the run. The hydrograph at the top of Fig. 2a also includes the average historical discharge calculated from hydrometric data from 1955 to 2013. Comparison of the sampled year discharge versus average historical values indicates a number of unusual, significantly higher discharge, flushing events from October through to the more normal freshet, and one flushing event just before the project started. In mid-July, 2 weeks prior to the first sample being collected, a rain event caused river discharge to increase from 23 to 54 m^3^ s^−1^ in one day (not shown in Fig. 2a). Following this July storm, river levels remained low and relatively stable (5–10 m^3^ s^−1^) through peak-spawn. Discharge spiked again twice during the post-spawn period. The first began in early October, when high amounts of rain and snow generated a steady increase in river discharge through to the first week of November (11 to 49 m^3^ s^−1^). Therefore, the final 2 weeks of the spawn season, when more than 50% of sockeye in the river were decaying, occurred during an ascending, flushing limb of the hydrograph, which is not normal for this time of the year. The second post-spawn flushing event occurred between January and February of 2015 when unseasonably warm weather generated snowmelt, causing river discharge to rise from approximately 10 to 52 m^3^ s^−1^ s. This mid-winter flushing event is historically not typical for this river, but could become more common with the changing climate regimes. The spring freshet began in late April (although valley bottom melt started in late March) and peaked on May 30 with discharge increasing from 10–20 to 91 m^3^ s^−1^. From May 14 to June 11, 12 bulk suspended sediment samples were collected (red dots in Fig. 2a), as the flows were too strong and the water too deep to access the bottom sampling sites safely. By mid-June the river level was low enough to resume the collection of bed sediment samples.

The low values for the g m^−2^ of sediment and the elevated values for floc factor at all sites pre-spawn were consistent with the post-scour transport and deposition of aggregated sediment and organic matter following the July storm. During peak-spawn water temperatures were warm (15 °C) and discharge was low and steady (Fig. 2a). During this time, however, the floc factor spiked again at the three most downstream sites, notably so at S-154 and S-200. These sites are both located downstream of high value spawning areas, but S-154 is in a reach with cobble too large for sockeye to spawn in (Kondolf and Wolman 1993). Although there was a significant relationship between the sediment floc factor and the total sediment nitrogen (µg) during all seasons except pre-spawn, the steepest slope occurred during the spawn (Fig. 2b).

**Fig. 3:**
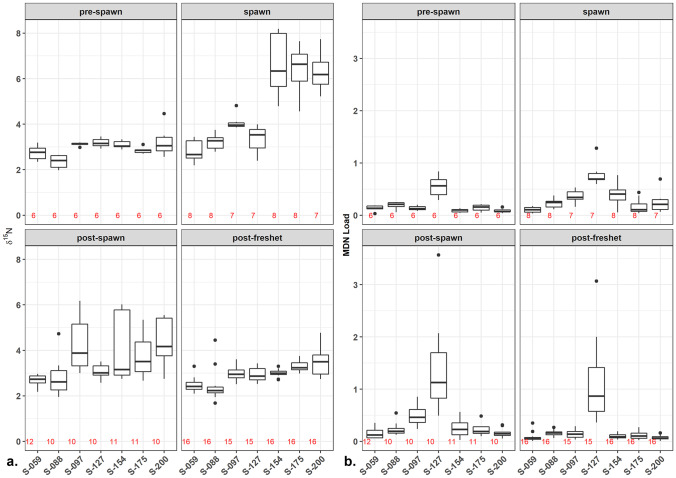
**a** Boxplots of sediment δ^15^N for each site by season. **b** Boxplots of MDN load (g m^−2^ of.^15^N) for each site by season. Numbers in red on the bottom of the graphs represent the number of observations per boxplot (*n* value)

During the spawn, sediment δ^15^N across all sites below the falls (i.e., where salmon were present) increased from 2.97 ± 0.43‰ (mean ± SD) pre-spawn to 4.67 ± 1.55‰ peak-spawn, and then peaked at 5.33 ± 1.61‰ in mid-October during peak-decay just before discharge began increasing (Figs. 2c and 3a). Sediment δ^13^C followed a similar pattern to the sediment δ^15^N before, during, and after the spawn (Fig. 2c). The C:N ratio also responded during the spawn, but inversely to δ^15^N due to the relatively higher contribution of N during the spawn and pre-spawn periods. Above the waterfall (S-059) bed sediment and MDN trends remained stable throughout the spawn event (pre-spawn δ^15^N = 2.74 ± 0.32‰; peak-spawn δ^15^N = 2.70 ± 0.40‰; peak-decay δ^15^N = 2.39 ± 0.30‰) suggesting it provides a suitable MDN background value for this environment.

Post-spawn, sediment characteristics again exhibited post-scour and deposition patterns due to the two hydrological flushing events (i.e., November 2014 and January 2015) that occurred between the spawn and the freshet. Post-spawn samples include three sample events in 2014 after the spawn, as well as three early spring/pre-freshet bed sediment samples collected between mid-March and late April 2015. Following these two high flow events the amount of bed sediment and MDN riverbed load increased at the three depositional sites, S-059, S-097, and S-127, although the only site where sediment δ^15^N and MDN load increased and remained elevated through the winter months following the November storm was at S-097. The average sediment δ^15^N following the post-spawn November flush was 4.33 ± 1.24‰, and this average further decreased to 3.21 ± 1.24‰ by late March (Fig. 2c).

**Fig. 4:**
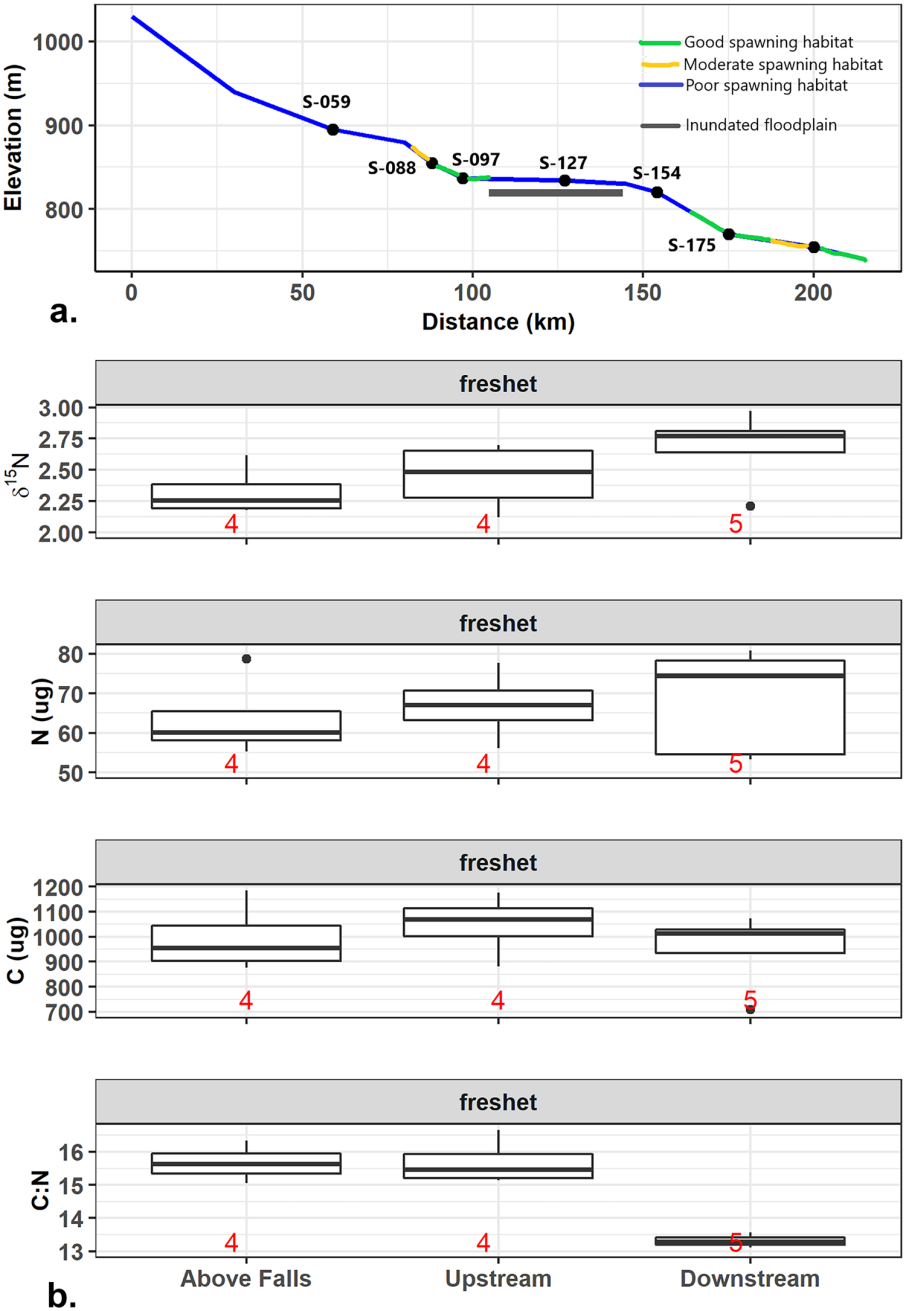
**a** A hypsograph for the Horsefly River that includes location of spawning habitat quality. Each black point along the slope line of the hypsograph represents the location of a sample site. The location of the seasonally inundated floodplain is indicated by the gray horizontal bar. **b** Boxplots showing the differences in δ^15^N, total nitrogen and carbon, and the C:N ratio of bulk suspended sediment collected during the freshet above the falls (S-059), and above (S-088, S-097) and below the inundated floodplain (S-154, S-175, and S-200). S-127 was not sampled during the freshet due to the lake-like feature and water depth in this region at that time. Numbers in red on the bottom of the graphs represent the number of observations per boxplot (*n* value)

Suspended sediment collected during the freshet had a lower average δ^15^N of 2.5 ± 0.28‰ than any bed sediment sample collected at any of the sites (red points in Fig. 2c). Like the stored bed sediment, the suspended sediment composition varied along the river length. The most notable spatial difference was in the C:N ratio between the suspended sediment collected above and below the inundated floodplain (Fig. 4b). Although the relative values of δ^15^N are low compared to the spawn, this pattern of increases accruing with travel distance is suggestive of overwinter storage of MDN with cumulative downstream transport. Finally, post-freshet or during the summer of 2015, the sediment δ^15^N was similar to 2014 pre-spawn sediment values (average δ^15^N = 3.02 ± 0.54‰ and average δ^13^C =  −25.39 ± 0.88‰). MDN loads at all sites except depositional reach S-127 were at or below values at the start of the project in August 2014.

### Spatial and temporal analysis of bed sediment

Linear mixed-effects models, using sediment δ^15^N and MDN load as the response variable, found that season was the most significant predictor of sediment δ^15^N and MDN load at all sites (Table 4). Site, however, as a random effect, accounted for the highest amount of variability within both response variables (marginal versus conditional R^2^ values in Table 4). However, the strongest model included the interaction between season and site (AIC values: δ^15^N ~ season =  −571.99; δ^15^N ~ season: site = -688.71; MDN load ~ season = 115.91; MDN load ~ season: site = 81.573). *P*-values for the models were calculated using a modified post hoc pairwise Tukey test (Searle et al. 1980). Sediment δ^15^N and MDN load were both significantly different between pre-spawn and spawn (δ^15^N: *p* < 0.0001; MDN load: *p* = 0.034). Sediment δ^15^N was also significantly different between spawn and post-spawn (*p* = 0.001), spawn and post-freshet (*p* < 0.001), and post-spawn and post-freshet (*p* = 0.024). MDN load was significantly different between spawn and post-freshet (*p* < 0.001) and post-spawn and post-freshet (*p* < 0.001), but not between spawn and post-spawn (*p* = 0.911). Neither sediment δ^15^N nor MDN load values were significantly different between pre-spawn and post-freshet (δ^15^N: *p* = 0.999; MDN load: *p* = 0.942). A series of models were run to examine sediment δ^15^N and MDN load, from the sites below the falls, as a function of sites grouped by morphology and sites grouped by substrate size. Annually, morphology was not found to be a strong predictor of sediment δ^15^N and MDN load (marginal R^2^ < 0.05, or 5%). Substrate size, however, was estimated to account for 42% of variability within MDN load. The modified Tukey test found significant differences in substrates between sand and all other substrate sizes (coarse gravel: *p* < 0.001; medium gravel: *p* < 0.001; and medium cobble: *p* = 0.022). Within each season, the models with morphology were stronger (i.e., lower AIC values), but the R^2^ values suggested that substrate size as an explanatory variable predicted more of the variability, especially for MDN load. A modified Tukey test for the seasonal models did not help explain trends beyond the models with individual sample sites. The only substrate that was consistently significantly different from the other substrates for MDN load was sand (*p* = 0.01 to < 0.001), which was only found at depositional reach S-127. During the post-spawn, medium gravel was a significant factor (*p* < 0.005) at depositional reach S-097. These are also the only two sites below the falls with a pool morphology which enhances deposition. Pools were significantly different for MDN load from both eddies and runs during the spawn (*p* ~ 0.05) and post-freshet (*p* < 0.003). There were no significant or notable differences in sediment δ^15^N concentrations between morphologies or substrate sizes during any of the seasons (*p* > 0.2).

**Table 4:** Summary table from linear mixed-effects models with season as the explanatory variables on sediment δ^15^N and MDN load. δ^15^N data was log base 10 transformed. Pre-spawn was set as the intercept value in each model with season, and S-059 for models with site. Summary table values are from R package sjPlot

**Predictors**	**Log10 (δ**^**15**^**N) ~ season**			**Log10 (MDN load) ~ season**		
	**Estimates**	**Confidence interval**	***p***	**Estimates**	**Confidence Iinterval**	***p***
**(Intercept)**	0.46	0.39–0.54	< 0.001	−0.87	−1.15 to −0.58	< 0.001
**Season (spawn)**	0.18	0.11–0.25	< 0.001	0.23	0.06–0.40	0.007
**Season (post-spawn)**	0.07	0.00–0.13	0.035	0.28	0.12–0.44	< 0.001
**Season (post-freshet)**	0	−0.06 to 0.06	0.949	−0.04	−0.19 to 0.11	0.572
**Random Effects**						
***σ***^**2**^	0.01			0.07		
***τ***_**00**_	0.00_Date_			0.01_Date_		
	0.01_Site_			0.12_Site_		
**ICC**	0.57			0.63		
**N**	7_Site_			7_Site_		
	21 _Date_			21_Date_		
**# of obs**	279			276		
**Marginal *R***^**2**^**/conditional *R***^**2**^	0.258/0.683			0.099/0.667		

Across all seasons, site as a random effect was estimated to account for approximately 40% of variation within sediment δ^15^N and nearly 70% of variation within MDN load (Table 4). Models were run with site as a fixed effect to further examine patterns within sites that emerged from the morphology and substrate size models.

During the spawn and post-spawn, sediment δ^15^N at all downstream sites and S-097 was significantly different than S-059 (*p* < 0.0001). At the sites below the falls during the spawn, sediment δ^15^N was significantly lower at the three most upstream sites (S-088, 097, and 127) compared to the three most downstream sites (S-154, 175, and 200) (*p* < 0.0001) (Fig. 3b). During the spawn, only the MDN loads at S-097, S-127, and S-154 were significantly greater than S-059 (*p* < 0.0001, Fig. 3b). MDN load at S-127 was also significantly higher than all other sites during post-spawn and post-freshet seasons (*p* < 0.0001). Post-spawn sediment δ^15^N values at S-097 were significantly different from S-059 (*p* = 0.009) but were not significantly different from any of the further downstream sites, although Fig. 3a shows that the highest variability in sediment δ^15^N values occurred post-spawn. Again, during post-freshet, there was a significant difference between sediment δ^15^N values at S-059 and all sites downstream of S-088.

**Fig. 5:**
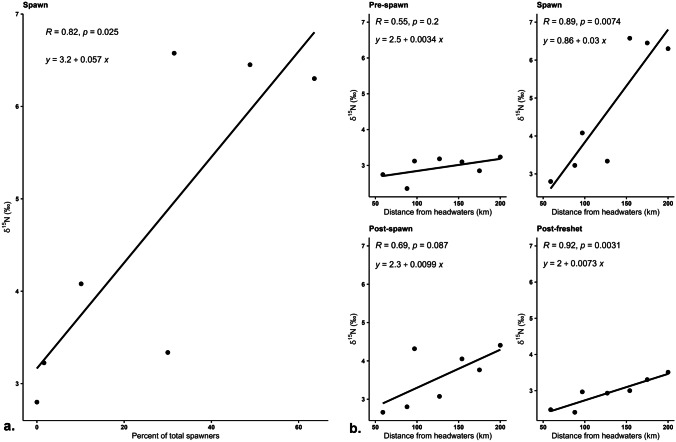
**a** Regression plot of cumulative percent of total upstream spawners in relation to average sediment δ^15^N at each of the sample sites, including S-059 above the falls, during the spawn period. **b** Regression plots showing the correlation between sediment δ^15^N and distance from the river headwaters at each of the sites during each season

The regression line for the percent of total upstream spawners plotted against average sediment δ^15^N during the spawn showed a significant correlation (*p* = 0.025) and an increasing trend in average sediment δ^15^N as percent of spawners increased (Fig. 5a). When average sediment δ^15^N at each sample site was plotted against distance from headwaters during each season there was also an increasing cumulative effect trend (Fig. 5b), with a significant correlation during both the spawn (*R* = 0.89; *p* = 0.007) and post-freshet (*R* = 0.92; *p* = 0.003) seasons. The slope, however, was much steeper for δ^15^N sediment concentrations sampled during the spawn due to a more substantial difference between the MDN signal in the upstream and downstream sites.

### Composition of sediment-associated organic matter

**Fig. 6:**
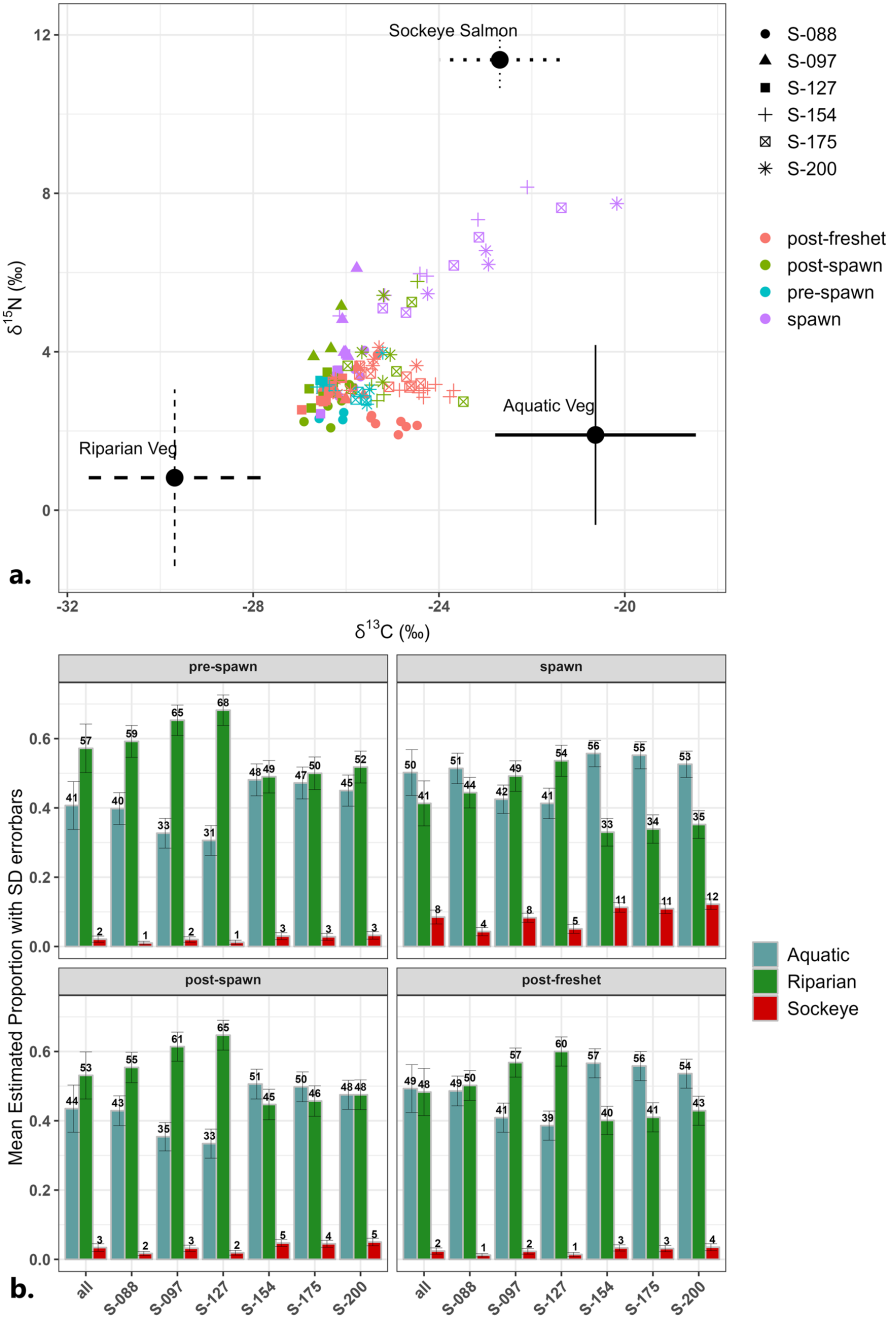
**a** Isospace graph of relative dual-isotopic signatures for the fine sediment and source materials. *X*- and *Y*-axis are δ^13^C (‰) and δ^15^N (‰) values for sediment sampled below the falls and all source material collected from above and below the falls. Error bars on source material represent 2× the standard deviation to account for natural variation. **b** Model outputs of the mean estimated proportion of each source at each of the below falls sites, and a column for the overall combined values, during each of the four seasons. Error bars represent the mean ± the standard deviation. Text labels above bars are the model estimated percent of contribution of each source

The isotope signatures of the sediment were also used to model the proportional contribution of MDN to the surficial fine bed sediment using the Bayesian mixing model MixSIAR. Bed sediment from each of the six sites below the falls was grouped into four seasons, thus resulting in a total of 24 sediment groups. The δ^15^N and δ^13^C values of the three source material groups (riparian vegetation, aquatic vegetation, and sockeye salmon) and 24 sediment groups are plotted together in Fig. 6a. This figure maps the dual-isotopic signature of the sediment from each season along with the source material to provide a visual of the sediment isospace relative to that of each source. This figure is important for showing that the sediment isospace falls within the bounds of the selected source materials. If the sediment had isotope values that fell outside of the source material isospaces, then it would have indicated that our sediment mixture included a source, or sources, not represented in the model. The model was initially run with four source groups where riparian vegetation was split into woody leaf litter and non-woody vascular plants. These two riparian vegetation sources shared a similar isospace in the bottom left of the biplot. Although the results were very similar between the three and four source models, the model with three sources resulted in less uncertainty according to size of error bars and model diagnostics. Along with testing the source-sediment isotope distribution, the isotope biplot in Fig. 6a is also helpful for interpreting the model estimated source contribution results shown in Fig. 6b.

The results from MixSIAR were consistent with the spatial and temporal differences found in the linear mixed-effects models. Figure 6a shows that the sediment isospace during most of the year is located in between the two non-salmon vegetation sources. During the spawn, there was a notable shift in the sediment isospace up and to the right towards the sockeye salmon isospace. Figure 6b is a series of bar graphs showing the MixSIAR model outputs of the estimated mean proportions of the sources for the sediment. The model outputs confirm that the vegetation source groups make up the largest proportion of the sediment-associated organic matter throughout the year. During the spawn, however, the model estimated that proportional contributions of salmon shifted from 1–2% pre-spawn to 4–12% during the spawn. Like the results from the mixed-effects models, we see a decreasing trend in the contribution of salmon to the sediment as the river discharge increased and the salmon carcasses were consumed or flushed downstream into the lake. Post-spawn the model estimated that the overall contribution of salmon dropped down to 2–5%.

The notable spatial difference between the three upstream (S-088, S-097, and S-127) and the three downstream sites (S-154, S-175, and S-200) found in the mixed-effects model was also captured by MixSIAR. The estimated proportions of salmon contributions were consistently similar between the three upstream sites, and between the three downstream sites. (Fig. 6b). In regard to the non-salmon sources, whether aquatic vegetation or riparian vegetation was the dominant source varied by site and season. During the spawn and post-freshet aquatic vegetation was strongly dominant at the three downstream sites, and riparian vegetation was strongly dominant at the three upstream sites during pre-spawn and post-spawn, which could reflect the flushing of headwater material during high flow events.

Seasonally, the vegetation contribution during the spawn and the post-freshet was similar at all sites. Aquatic vegetation and salmon have a similar δ^13^C isospace but have very different δ^15^N isospaces. During the spawn when the sediment shifted up towards the nitrogen isospace of salmon, it also shifted right towards both the carbon isospaces of salmon and aquatic vegetation (Fig. 6a). The inclusion of concentration dependence in the model corrected for different C:N ratios between the salmon tissue and the aquatic vegetation and therefore more of the sampled sediment contribution was estimated to be from the aquatic vegetation (~40- to 60%) rather than the salmon (4–12%) during the spawn. Models run without correcting for concentration dependences estimated the proportional contribution of salmon to be closer to 50% at the downstream sites.

**Fig. 7:**
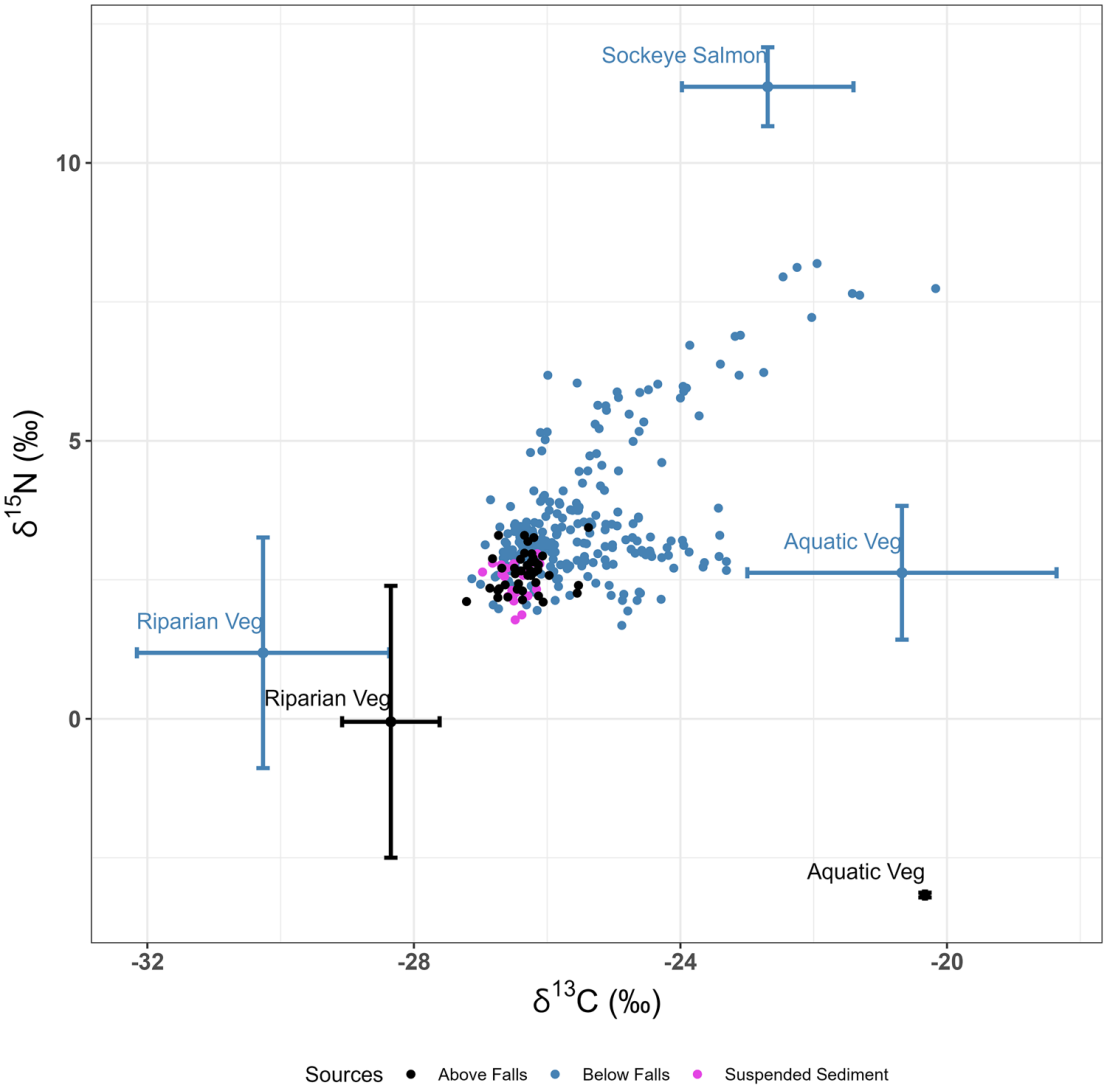
Graph of relative dual-isotopic signatures for fine bed sediment above and below the falls and bulk suspended sediment. Source material sampled both above and below the falls is separated out and grouped into the same color scheme as the sediment (i.e., blue dots and blue source errors bars are for below falls sediment, and black for above the falls). Seasonal groupings were left off for a better visual comparison of bed sediment from below and above falls. All bulk suspended sediment was collected during the freshet and are shown in pink with no source material sampled at that time. Error bars on source material represent 2× the standard deviation

The relative isospace for the above falls bed sediment and source material were plotted alongside the below falls bed sediment and source material for comparison in Fig. 7. During the freshet, the suspended sediment isotopic signatures sampled below the falls were similar to the pre-spawn and post-freshet bed sediment signatures, and the year-round signature of the sediment sampled above the falls at S-059 (Figs. 6a and 7). The distribution of the above falls bed sediment shown in Fig. 7 suggests that riparian vegetation rather than aquatic vegetation was consistently the dominant contributing source, with a shift towards aquatic vegetation occurring in the summer months (pre-spawn/post-freshet) as well as during the spawn in the late-summer and autumn months (three black dots at ~-25 δ^13^C in Fig. 7). The relative isospace for riparian vegetation collected above and below the falls were quite similar. Riparian vegetation samples from above the falls were divided into riparian herbaceous and leaf litter to investigate the potential source of higher δ^15^N values in the above falls bed sediment (not shown in Fig. 7). This showed that the elevated δ^15^N values was likely from common horsetail (*Equisetum arvense*). The relative δ^15^N of the aquatic vegetation from above the falls, however, was substantially lower in δ^15^N than from below the falls.

## Discussion

While there has been a lot of previous research looking at the relationship between fine sediment and anadromous spawning salmon in freshwater streams, this project is one of the few to assess the broader spatial (Ruegg et al. 2020) and temporal (Holtgrieve et al. 2010) context of MDN transfers over a hundred kilometers in a downstream direction toward the river outlet and across the annual hydrograph, and appears to be the first to do this on an interior stream. Sampling approximately 130 km of an interior river channel across multiple seasons allowed us to explore the downstream movement of sediment-associated MDN and how river morphology and hydrologic variation interact to influence sediment and nutrient storage. By using a Bayesian stable isotope mixing model, we were able to estimate the MDN contribution within the context of other natural non-salmon related nutrient sources and cycling.

High productivity months in interior streams are typically June or July (depending on spring freshet timing) to September when river levels are low, water temperature is elevated, and day length is long. The addition of new MDN from September spawns and autumn die-offs would contribute soluble MDN to downstream nursery lakes with particulate MDN potentially retained in reaches of the channel bed. MDN that is stored over the winter can be re-mobilized and distributed during the spring freshet and contribute to downstream productivity. In-stream storage duration is partly a function of water residence time in retention zones (Zarnetske et al. 2011). Certain physical attributes within a stream, such as deep pools and log jams, facilitate longer term retention of nutrients. During the 2014 spawn in the Horsefly River, the sediment characteristics were found to follow patterns expected from previous research on both salmon effects and nutrient cycling in interior streams. This study, as with other published findings, identified a high degree of spatial variation in response to the physical, chemical, and biological impacts of spawning salmon (Janetski et al. 2009). Substrate size, stream temperature, discharge, and numbers of upstream spawning salmon are all variables that have been identified as regulating stream response to salmon disturbance and MDN enrichment (Wipfli et al. 1999; Chaloner et al. 2007; Moore et al. 2007; Tiegs et al. 2008). As expected, the movement of MDN and non-salmon nutrients in the Horsefly River across all seasons was found to be influenced by interactions of discharge, channel morphology, substrate size, and number of upstream spawners. This project, however, found that the majority of significant differences observed in the data analyses corresponded with a downstream accumulation/magnification of MDN due to a longitudinal gradient of varying river morphologies, and the subsequent spawning habitat quality. Although individual site morphology and substrate size were important, there was a consistent pattern between the MDN signal in the stored fine bed sediment and the cumulative effect of distance (Figs. 3a, 4b, and 5b). Vanotte et al. (1980) suggested that downstream communities are structured to make use of upstream nutrient waste. Re-suspended sediment and dissolved and particulate nutrients from spawning salmon are transported with flow downstream from upstream spawning activities but can be delayed from leaving the system due to a variety of internal stream processes and functions.

During the spawn, trends in sediment characteristics along with correlations between the floc factor and the sediment-associated nitrogen provided strong evidence that fine sediment re-suspended by spawning salmon can aggregate with MDN and re-settle downstream in the top 10 cm of the bed sediment and associated biofilm (Fig. 2b). Soon after salmon arrived in the Horsefly River the re-suspended surficial fine bed sediment δ^15^N began increasing and the C:N ratio began decreasing at all sites below the falls, a natural salmon barrier, and continued to do so through the peak-spawn and decay, especially so at the three most downstream sites. Rex and Petticrew (2010) and Albers and Petticrew (2013) found that in a flume and a spawning channel study, MDN-sediment flocs were transferred downstream to the streambed in the presence of live and decaying salmon. Albers and Petticrew (2013) reported a positive correlation between the increase in δ^15^N values of sediment infiltrated in the gravel bed and the number of spawning salmon. The amount of sediment-associated MDN transported out of a river reach is regulated by the quality of the contributing habitat (substrate and flow conditions), which also influences the number of spawners supplying MDN and re-suspended sediment. Locally at a site, however, the substrate and flow conditions regulate the ability of that channel reach to trap/settle out the delivered suspended MDN load.

The regression plots for cumulative numbers of upstream spawners and average sediment δ^15^N during the spawn (*p* = 0.025) showed a statistically significant relationship (Fig. 5a). The linear fit was strongest for the three most upstream sites (S-059, S-088, and S-097), but became less so at S-127. The average sediment δ^15^N at S-127 was lower than expected for the upstream spawn numbers, whereas the δ^15^N at S-154 more than doubled when cumulative upstream spawner percentages only increased by ~1.5% between those sites. The last two downstream sites, S-175 and S-200, exhibited high δ^15^N which supported the correlation pattern but they did not continue to increase as substantially as expected given approximately 70,000 more spawners were counted between S-154 and S-200. Upstream spawner numbers contributed to the longitudinal pattern among sites, but did not solely explain all observed spatial differences. Tiegs et al. (2008), Holtgrieve et al. (2010), and Bellmore et al. (2014) each found that the size of the streambed substrate was a controlling factor in the response of biofilm to spawning salmon. Bellmore et al. (2014) concluded that the stream biofilm was more likely to be enriched if the substratum of the streambed was not suitable for spawning (i.e., too coarse), suggesting that biofilm can trap organic particles and remains undisturbed by spawners. Sites S-059, S-088, S-175, and S-200 were all classified as coarse gravel sites; S-097 was a medium gravel site, S-154 was medium cobble, and S-127 was sand (Table 1). S-154 was the furthest upstream of the three sites in the downstream grouping and the only downstream site that had no active spawners because the cobble is too large for sockeye (*D*_50_ = 110 cm). S-154 also stood out from the other downstream sites by having the highest total grams of fine sediment, MDN load, and floc factor values during the spawn indicating its nutrient storage and retention capacity and corroborating the findings presented by Bellmore et al. (2014).

At all sites during the spawning period Spearman Rho correlations found that the floc factor was positively correlated with sediment OMR (*r* = 0.48; *ρ* = 0.0004), and negatively correlated with the C:N ratio (*r* =  −0.46; *ρ* = 0.0006) supporting the linear model regression lines in Fig. 2b showing the influence of nitrogen supply, including MDN, on flocculation. Near the middle of the peak-spawn, the floc factor increased at the three downstream sampling sites (Fig. 2a). The highest values observed for floc factor were from S-154 (Fig. 2a). These results support the spatial hypothesis proposed by Holtgrieve et al. (2010) that large substrate (> 110 mm) is less vulnerable to benthic disturbance by salmon. Furthermore, Albers and Petticrew (2013) found that undisturbed biofilm can act to trap sediment-associated MDN from upstream spawners in interior B.C. streams. Our results provide evidence that the undisturbed substrate interstitial space and biofilm on the larger cobble at S-154 did act to trap upstream MDN. The MDN load (g of ^15^N m^−2^) in the bed sediment and biofilm continued to increase at S-154 after peak-spawn (from 0.15 g m^−2^ to a max of 0.6 g m^−2^) and remained elevated during the entire time decaying salmon were present. These elevated values, however, did not persist beyond the first post-spawning hydrologic flushing event (0.24 g m^−2^).

Holtgrieve et al. (2010) also reported that enriched biofilm δ^15^N in an Alaskan stream had returned to pre-spawn levels by the following spring (i.e., post-spawn to freshet), and surficial biofilm was likely not a long-term storage option due to fluctuating hydrological and geomorphological conditions. However, coastal watersheds do not typically have large mid-stream seasonally inundated floodplains that can act as large sinks of both particulate and soluble nutrients, and typically coastal systems experience high flows in the autumn months, when interior streams generally have low flows. In other sampling undertaken on the Horsefly River that occurred concurrently with this project, pulses of ammonium and total dissolved phosphorus were found to be moving through the floodplain riparian hyporheic zone during the 2014 spawn indicating a possible pathway for longer term MDN retention (Rasmus 2017). Stored surficial sediment was sampled along the floodplain meanders at S-127 and at the upper reach of the floodplain at S-097. Good spawning habitat was observed at S-097, but it was the only site sampled that had a deep, side-channel pool. Patterns in MDN load at these two depositional sites suggest that they not only increased the chance that suspended MDN would settle out of the water column, but they also would exhibit lower velocities at the sediment–water interface than the shallow cobble bed at S-154. Ruegg et al. (2020) found that during a salmon run pools had the greatest biofilm biomass compared to edge and riffle-run stream morphologies. Although MDN stored in the surficial biofilm at S-154 was likely scoured during the late-autumn storm, MDN load at S-097 and S-127 either increased or remained elevated, following the post-spawn and mid-winter flush. These results suggest that in these pool environments either further deposition of sediment-associated MDN occurred following each high discharge event, or that the MDN stored in these locations is being affected by in-bed processes of enrichment by bacterial ^14^N uptake (Pinay et al. 2003).

These findings indicate that a combination of good spawning habitat interspersed with large substrate and depositional morphologies may act to retain and utilize nutrients within a river across a range of temporal periods, which is especially apparent in the downstream reaches (Figs. 3 and 4). Verspoor et al. (2010) studied phosphorus levels and stable isotopes in biofilm from 24 interior streams in north-central B.C. and found evidence that primary productivity increased due to an accumulation of MDN from multiple spawning years. In our study, the C:N ratios of the bed sediment at the three most downstream sites were the only ones which remained below 12 throughout the full sample year (Fig. 2c). It is therefore possible that the MDN associated with stored fine sediment and biofilm during the spawn was taken up by benthic biota (Rinella et al. 2013) and/or transported into deeper subsurface/hyporheic zones for longer storage, rather than being flushed completely during the November 2014 and January 2015 storms. Chaloner et al. (2007) and Tiegs et al. (2008) both found that biofilm productivity increased with MDN enrichment. Albers (2011) found that during the spawn biofilm composition shifted from algal (autotrophic) dominance to bacterial (heterotrophic) dominance. O’Keefe and Edwards (2002) and Pinay et al. (2003) found a rapid uptake and transformation of salmon-derived nitrates in the riparian and streambed hyporheic zones. The isotopic signatures of the non-salmon sources collected along the Horsefly River show a notable difference in the nitrogen isotopic values but not carbon. For example, the signatures of aquatic vegetation (vascular macrophytes) which is able to utilize salmonid MDN, differs significantly depending on whether it was sampled above or below the natural salmon barrier (above falls average: C:N = 40.65 ± 0.5, δ^15^N =  −3.17 ± 0.04‰, δ^13^C −20.33 ± 0.01‰; below falls average: C:N = 13.15 ± 1.89, δ^15^N = 1.90 ± 0.57‰, δ^13^C −20.63 ± 0.54‰) (Figs. 2b and 7). These averages represent a relatively small sample size (above falls *n* = 2; below falls *n* = 12) but do show another piece of supporting evidence of an MDN legacy being utilized by primary producers. Harding et al. (2004) also found that submersed aquatic macrophytes in ponds with coho salmon (*Oncorhynchus kisutch*) had enriched δ^15^N compared with ponds that had no coho present. Figure 7 shows the complexity of tracing MDN throughout a relatively large natural interior river, one with a watershed that provides a continuous source of headwater organic matter alongside that provided by the annual sockeye run.

Around the same time the Horsefly River received a pulse of MDN from the sockeye salmon, it also received nutrients from decaying riparian and aquatic vegetation. Streams in the Central Interior of B.C. receive nutrient inputs from the riparian zone as litter during the autumn, from terrestrial and bank soil organic matter during storm events throughout the year, but especially during the spring snowmelt and subsequent flooding. The results from MixSIAR, a Bayesian mixing model, further supported the spatial and temporal findings from the sediment trends (Fig. 2) and the mixed-effects models (Table 4). MixSIAR estimated that there was a notable short-term temporal response in the bed sediment to the presence of spawning salmon, and that this response varied notably between the sites upstream (weaker) and downstream (stronger) of the floodplain (Fig. 6). During the spawn the proportional contribution of salmon to the bed sediment increased by 8–9% at the downstream sites and 3–7% at the upstream sites. The C:N ratio provided evidence of a cumulative effect of nutrients being retained and utilized down the river, in that upstream sites received more allochthonous inputs (C:N > 15) and the downstream sites received more autochthonous inputs (C:N < 15) (McConnachie and Petticrew 2006). MixSIAR results support this with a higher estimated contribution of salmon decay products and aquatic vegetation at the downstream sites versus riparian vegetation at the upstream sites. The only other study that used a stable isotope mixing model to determine estimated salmon contributions to bed sediment was McConnachie and Petticrew (2006). They ran a similar model using IsoSource to compare salmon with terrestrial and algal sources and found that during the spawn salmon were estimated to contribute close to 50% of the sediment-associated organic matter. When running our model without correcting for concentration dependence the MixSIAR results were similar to the IsoSource results. The concentration-independent model estimated that the proportional contribution of salmon increased from 4–9% pre-spawn to 23–50% during the spawn. Both MixSIAR and IsoSource found that salmon-associated nutrients contributed proportionally very little to freshet sediments. There has been some debate around correcting for concentration dependence when using sediment as the consumer, but in controlled experiments Phillips and Koch (2002) found that increasing or decreasing the *N* concentration of just one source drastically adjusted the model estimates of proportions. Due to the significant difference between the C:N ratio of salmon tissue versus vegetation, it was decided to correct for concentration dependences in our models. However, salmon tissue shares a similar carbon isospace with aquatic vegetation. During the spawn, there was a clear and strong shift in the bed sediment signal that moved the values about halfway between salmon and aquatic vegetation (Fig. 6a). The concentration corrected model estimated that the shift was due to a larger increase in contribution of aquatic vegetation than salmon at the downstream sites, which is seasonally very probable, but also possible that the model underestimated the contribution of salmon. Verspoor et al. (2010) used a very basic two source mixing model without the capability of correcting for concentration dependences and found that salmon at most contributed 22% to biofilm in 24 interior streams. They felt that this number was underestimating the contribution, but perhaps we tend to want to overstate the short-term salmon enrichment effects given that salmon represent the original marine source for these systems. Stable isotope mixing models have the potential to increase our understanding of the role of salmon in freshwater nutrient cycling, but further research into improving accuracy, or our faith in the accuracy, of the results is needed.

## Conclusion

In conclusion, the analysis of sediment trends, the linear mixed-effects model outputs and the MixSIAR models all indicated that (1) there was evidence that flocculation between sediment and suspended organic matter, especially during the spawn, was occurring but; (2) upstream spawner numbers, substrate size, stream morphology, and discharge all appeared to be relevant to both flocculation, the enrichment magnitude and the retention time of sediment-associated MDN; (3) the MDN isotopic signal in the stored surficial bed sediment in this sample year was short term, which we presume was somewhat regulated by unusually high pre-freshet flows; and (4) there was a significant longitudinal spatial distinction between the sediment-associated MDN at sites upstream and downstream of the freshet-inundated floodplain. Determining the length of time MDN is retained is complicated by the challenge of distinguishing MDN from the current year’s salmon with channel-stored MDN from previous years, and current MDN versus past season’s MDN taken up by other sources (i.e., riparian and aquatic vegetation) now moving as particulate organic matter in the stream. Addressing these distinctions is important for improving stream management within the context of declining salmon populations and changing hydrological regimes, which are both responding to climate changes and anthropogenic disturbances. Another key aspect of managing salmon and non-salmon resource subsidies is the importance of differing scales of response to spawning salmon (Janetske et al. 2009; Verspoor et al. 2010). While most previous studies have focused on MDN transport and storage over relatively small scales and time periods, the findings from this study indicate a strong difference between sediment-associated marine and terrestrial/freshwater organic matter between upstream and downstream sample sites. While the 130 km river section studied over a year is novel to this project, it is also important to recognize that the conditions in interior streams differ significantly from coastal systems. Interior stream environments are fundamentally different in terms of the hydrologic regimes and spatial expanse. Interior streams may have a greater capacity to store MDN because the timing of the salmon runs correspond to the timing of local vegetation die backs and their nutrient inputs which can reflect past years’ MDN uptake but also simply because they are so far removed from the marine origins resulting in the high-quality nutrients being integrated into the less productive interior ecosystems quickly. This research is beneficial for future salmon habitat and stream ecosystem restoration activities on interior streams, especially as these activities typically focus solely on isolated spawning habitat and the negative aspects of fine sediments over their valuable role in nutrient distribution and storage.

## Data Availability

All data used in this publication is available upon request from the corresponding author.
